# Mindfulness Meditation as Psychosocial Support in the Breast Cancer Experience: A Case Report

**DOI:** 10.3390/bs12070216

**Published:** 2022-06-29

**Authors:** Letizia Iannopollo, Grazia Cristaldi, Caterina Borgese, Samuela Sommacal, Giulia Silvestri, Samantha Serpentini

**Affiliations:** Veneto Institute of Oncology IOV—IRCCS, 35128 Padua, Italy; letizia.iannopollo@iov.veneto.it (L.I.); grazia.cristaldi@gmail.com (G.C.); caterina.borgese@studenti.unipd.it (C.B.); samuela.sommacal@iov.veneto.it (S.S.); giulia.silvestri@iov.veneto.it (G.S.)

**Keywords:** breast cancer, Mindfulness, Quality of Life, well-being, depression, anxiety, self-esteem, coping strategies, adaptation, group therapy

## Abstract

In the last decade, Mindfulness-based interventions have been increasingly used in health care settings, particularly in the context of cancer. Research documents the efficacy of these interventions for decreasing the burdens of stress, anxiety, depression, fatigue, sleep disorders, and other symptoms. This article describes the case report of a patient with breast cancer, highlighting her personality, defense mechanisms, and traumatization connected with the disease. General information about the patient’s personal and medical history is presented in addition to the trajectory of psychoncological support, focusing on objectives, intervention strategies based on Mindfulness, and outcomes. The intervention is a combination of individual and group therapies, with particular reference to the use of Mindfulness in a group setting. The goal is to provide the patient with both a peer sharing experience as well as the tools to manage psychoemotional reactions through the development of awareness and a better relationship with herself. The main hypothesized consequences are an increase in self-esteem and coping strategies, which are necessary for a successful adaptation to cancer. The objective of the Mindfulness intervention is to promote the maintenance of an adequate Quality of Life (QoL) and psychological well-being, during and after treatment, transferring these skills into daily life.

## 1. Introduction

Current oncological care is characterized by innovative and personalized therapies which offer patients a better prognosis and Quality of Life [1]. This approach to oncology medical care also includes an interdisciplinary team to provide a more comprehensive approach which includes addressing the psychosocial needs of patients. Evidence that physical and psychological symptoms negatively affect emotional, social, and work functioning [1,2,3] is clearly established, and these decrements in functioning affect compliance, adherence to treatments, rehabilitation, and survival [4]. As one of the main objectives of multidisciplinary care is improvement of Quality of Life, psychosocial support is considered one of the fundamental elements of treatment. Within oncology care, the psychoncologist mediates between the medical team, the patient and the family, with the aim of promoting individualized care.

In order to mitigate the negative psychosocial impacts of cancer, multiple interventions can be proposed in different stages of the disease (prevention, diagnosis, active care, palliative care, and end-of-life). For example, numerous studies [5,6,7] report how group activities in various stages of treatment have different functions, such as facilitating identification with other group members, increasing the expression of emotional states, and implementing adaptation strategies to the disease. These processes are supported by mechanisms of attunement and emotional mirroring, which may help in forming relationships, particularly in a safe, nonjudgmental and mutually supportive environment [8].

Mindfulness is defined as a meditative practice and a state of awareness accompanied by a nonjudgmental attitude of acceptance and open-mindedness [8,9,10]. In the psychoncological context, several studies document the benefit of Mindfulness, which is useful for improving mood, sleep, psychosocial adaptation, coping strategies, and stress reduction. In particular, Schell and colleagues identified that Mindfulness interventions improve Quality of Life and sleep through a reduction in anxiety and depression, even six months after surgery [10,11]. Similar and equally significant results showed that after Mindfulness training, patients express, on the one hand, lower levels of affective symptoms (e.g., distress and a feeling of burden), and, on the other hand, new coping strategies and better survival outcomes [12].

The practice of Mindfulness produces positive effects for psychological well-being that last beyond the moment of formal meditation. Over the past three decades, meditation practices have been increasingly incorporated into psychotherapeutic programs [13,14]. A large body of research shows the effectiveness of these Mindfulness-based interventions in reducing symptoms such as anxiety [15], depression [16], substance abuse [17], eating disorders [18], and chronic pain [19], and in improving well-being and Quality of Life [20]. This change in resiliency after Mindfulness training is attributed to a process associated with perceptual change, in which there is greater awareness of personal thoughts and feelings, as well as the ability to redirect them with acceptance and stress reduction [21].

In light of several studies which identify Mindfulness as an effective intervention for patients with breast cancer, we applied these strategies to clinical practice in oncology care.

In particular, the main hypothesis of this case report is that individual psychological support should be associated with group therapeutic work, which includes psychosocial support. Through the association of these two interventions, patients may develop a new self-awareness, which promotes generalizable cognitive, behavioral, and emotional empowerment in their daily lives [22].

Consequently, training was proposed to patients being treated at the Veneto Institute of Oncology-IRCCS (IOV) of Padua that incorporated simultaneous individual and group interventions, with the latter focusing on Mindfulness. Thus, the present case report aims to describe the clinical path of a patient in the Breast Unit (IOV) in that combined intervention approach, to illustrate the implementation and effectiveness of this comprehensive intervention. To respect the patient’s privacy, an attempt was made to change information to preserve her anonymity.

## 2. Case Report

### 2.1. Personal History

B.A. is a 47 year old woman (at the time of cancer diagnosis), who lives in a small town in northern Italy, is married, and has two young sons. Her parents are alive and live near her; she has an older sister who provides care for her together with her partner (the primary caregivers). B.A. has a university education, which enabled her to pursue a career in healthcare; although her profession was an essential component of B.A.’s identity, after diagnosis she decided to take a leave from work. Another fundamental component that characterizes the patient is that she participated in competitive athletic events for many years. B.A., despite the difficult experience of illness, wishes to take care of her extended family, in the hope that all family members will be able to maintain the well-being and Quality of Life that belonged to them until now. Regarding her marital and family situation, B.A. reports a peaceful climate, good communication, and functional relationships. There are no economic or social difficulties, and she considers herself well integrated in the social context in which she lives.

### 2.2. The Therapeutic Group Path Based on Mindfulness Meditation Techniques

Mindfulness meditation practice aims to encourage people to develop a particular form of awareness by changing their view of experiences and events [23]. Mindfulness is described as “the ability to turn attention to yourself, in order to change the way you pay attention to the experience you are living: by assuming a compassionate attitude”. Great importance is given to attention, because it allows people to explore four areas of experience: sensations (body), thoughts (mind), emotions (mind-body), and behavior (action). In this way the usual experience of oneself is expanded. Typically, there is a misconception that important things happen at the level of mental (cognitive and emotional) experience, paying little attention to the sensory experience. However, Mindfulness practitioners assume [24] that Mindfulness meditation teaches and reminds people that the knowledge they have of themselves is the result of a complex psychocorporeal sense, which interacts with the environment [25]. People typically analyze and interpret these observations to give them meaning based on both their lived experiences and their social values. Thus, these experiences of daily life allow people to learn the main rules of adaptation, which are fundamental components for survival and pursuing goals, such as being healthy and happy, and having satisfying relationships. As human beings exist in increasingly complex social contexts, they have objectives that promote social survival, which allow them to build an identity that includes different social roles: daughter, mother, wife, worker, and so on. Therefore, people are motivated to develop a complex identity structure which instills balance and well-being, and is well integrated into the lives of other people in their social context [26].

Mindfulness meditation techniques focus on increasing awareness of these habitual patterns and assuming a new awareness in order to build a conscious and compassionate relationship with oneself and with the surrounding environment [22,27]. The ultimate goal of this philosophy of life is to raise awareness of the benefits acquired in everyday life in order to give everyone the opportunity to improve their relationship with themselves and to base their existence on unconditional acceptance of themselves by choosing how they want to live. Consequently, this Mindfulness-based lifestyle fosters collaborative relationships and limits destructive behaviors that are based on criticism and judgment. According to Mindfulness practice, change should start with the relationship one has with oneself and then extend to other relationships.

In Mindfulness therapy both theoretical and practical lessons are experimented with in a group setting and practiced at home to achieve cognitive and emotional empowerment. Through these techniques, the therapist promotes improvements in the patient’s self-esteem, a decrease in criticism and judgment towards herself, and, at the same time, recognition of her weaknesses and strengths [6]. Mindfulness meditation also supports patients’ development of a new relationship with cancer, by implementing an attitude of compassion and acceptance of traumatic experiences related to cancer [22]. Furthermore, this practice endorses effective and assertive communication with other significant people in order to obtain supportive, intimate, and satisfying relationships in the family and in social spheres. In this way, the patient has the opportunity to learn to manage the experience of loneliness and lack of communication associated with cancer [5].

### 2.3. Psychological Management

B.A. was contacted by the psychoncology service on the advice of her oncologist, because prompt psychoncological support was determined to be necessary to help her cope with newly diagnosed breast cancer. After three meetings, the psychoncologist administered three questionnaires (Distress Thermometer [28], Hospital Anxiety Depression Scale-HADS [29], and EORTC QLQ-C30 [30]), to obtain an objective assessment of the needs and difficulties that emerged during the interviews. Subsequently, the clinician proposed both individual therapy as well as a group intervention based on Mindfulness meditation techniques. The therapeutic hypothesis was that these two approaches would support B.A. in the management of intense emotional experiences, which she reported, and with facilitating her adjustment to symptoms of distress related to cancer.

The goal of combined treatment was to create conditions in which B.A. could share the traumatic issues that she described in the initial interview: anxiety, mood swings, insomnia, and identity traumas related to both her body (alienation from her body) and professional life (conflict about taking a leave from work). B.A. expressed emotional experiences of intense discomfort, loss of life trajectory, disorientation, uncertain future planning, and a clinical level of distress that was linked to the fear of both death and loss of family affections, accompanied by feelings of guilt.

The main objectives of the treatment were:

- To promote the establishment of a connection characterized by trust and empathy that would allow B.A. to view the therapeutic relationship as a safe place, and help her mitigate post-traumatic stress and the experience of threat.
- To make aspects of her personal and professional identity more explicit so that she could reimagine the identity structure that characterized her life before the illness. In this way, the clinician wanted to allow her to assimilate and transform the traumatic experience, reaching an appreciation for the evolution of her own identity and adaptive strategies.
- To participate in therapeutic Mindfulness meditation sessions together with other cancer patients in order to improve B.A.’s psychoemotional health and the quality of her life. Group sessions offered the opportunity to learn new behavioral, cognitive, and emotional skills. As reported in the literature, Mindfulness techniques nurture greater skills in self-reflection, understanding, control, and management of emotional and behavioral reactions [6].

### 2.4. Psychoncological Intervention Path

The treatment plan provided for weekly alternation of individual and group meetings throughout the course of active treatments (neoadjuvant chemotherapy, surgery, and radiotherapy). Meditation meetings were scheduled weekly, face-to-face or online (due to the COVID-19 pandemic); the group was made up of 12–14 patients, in active cancer treatment in either the follow-up or metastatic phase (not in the end of life phase). The group was made up of women with breast, ovary, lung, and melanoma cancer. The age of the patients ranged from 23 to 65. The limiting requirements for course participation were the absence of severe psychiatric pathologies, the ability to move independently, being able to phase simple movements, and being able to sit for the duration of the meeting.

The treatment proposed was intended to pursue the following issues:

- The psychoemotional state and social skills of the patient at the beginning of treatment (T0), in order to learn new skills of mentalization, self-acceptance, evaluation, and choice of new strategies, which are useful for reducing anxiety-depressive states, self-criticism, isolation, and insomnia [31],
- Aspects related to treatments and their side effects,
- Trauma resulting from body image after surgery,
- Relational and communicative aspects with other patients and family members, and within a social context after 6–8 months,
- Problems related to body rehabilitation, which is necessary to recover post-operative function (mobility and strength of the arm),
- Physical and emotional intimacy with the partner.

The ultimate and general objective of the treatment of cancer patients is to encourage the resumption of social and working life roles and to rebuild a new identity, which should be based on a path of awareness, acceptance, and personal growth.

The Mindfulness-based group intervention (Mindfulness-based living course, MBLC) is structured as eight meetings [31] during which patients learn to familiarize themselves with their internal operating models (IOM) [32], allowing them to internalize and stabilize ideas about themselves and the world around them. First, the therapist introduces the model theory, and then lets the patients put into practice the information learned through guided meditations and, finally, through moments of sharing and facilitating the experience.

The clinician initially encourages oncological patients to pay attention to the way their minds work, observing their thoughts, emotions, and behaviors, and recognizing how they react to experiences of rejection, shame, self-criticism, denial, estrangement, relentless search, and desire regarding personal experiences, acceptable or not. Guided meditation allows patients to experiment with self-reflexive methods that favor the empowerment of new skills and self-efficacy through group learning and reflection techniques implemented during the sharing phase of the practice. At the end of each meeting related homework is assigned to practice at home [31].

Effective ways to change habits are explored, based on calming cognitive-emotional activity and exploring bodily sensations; an important aspect of Mindfulness concerns learning to consciously listen to bodily sensations, relating them to psychophysical activity [31]. This particular aspect encourages an update of the self-awareness experience, which is relevant in responding to the daily tendency to minimize bodily experience, clouded by the activity of thought. During group activities, patients learn a new mind-body relationship in which the body’s experiences (sensations, perceptions, respiratory rhythm, tensions, body temperature, or the absence of conscious body experience) assume a reliable and safe support role, which allows them to accept and contain their experiences [24]. Thanks to this learning process, psychoemotional experiences can be managed and used to improve the personal experience of oneself (self-esteem, social, family, and work roles) through the instruments of self-acceptance and compassion [31,33].

Group participants usually become more familiar with the concepts of acceptance and compassion during individual psychoncological sessions. Through group activity, this knowledge is strengthened and stabilized allowing patients to reflect, experiment, and share these aspects with the peer-to-peer group [31].

Thanks to the group sharing of experience, patients assume an equal role among adults [26]; in this way, self-narration takes place using an adult self-image. This process empowers patients to be responsible and to experience themselves fully in body and mind, in a protected and nonjudgmental context which shapes their way of living and sharing their experience, with the guidance of the group leader. New learning within the group allows patients to socially experiment with a new self, helping them express how they feel, and observe how the context reacts to them [32].

After about four meetings, patients begin to internalize what they experience inside the group, thanks to alternation with individual therapeutic sessions. In these meetings patients experiment with their desire to experience a new self; they carry out functional self-reflection, comparing what they learned within the group with the experience of daily life (especially with family members and in the workplace). They focus part of their therapeutic work on the implementation of these skills [34] in order to improve self-esteem and their ability to face the daily challenges related to the experience of the disease, and also to project themselves toward new future possibilities [31].

Mindfulness techniques are based on training and reiteration of the meditative experience, increasing the level of autonomy from time to time; to this end, movements (such as walking and conscious movements) during meditation sessions are planned. In this way, the connection between group and daily life activities grows; the main goal is to encourage greater familiarity and confidence in the techniques learned, thanks to the benefits obtained. For this reason, in each meeting there are sharing moments dedicated to experiences outside of therapy during which everyone can express strengths or weaknesses of the practice of Mindfulness. A fundamental aspect of group sharing is that the leader helps the speaker retrace every aspect of the experience (sensory, emotional, cognitive, and behavioral) without judgment or interpretations. No one, not even the group leader, can judge the experience of others; the goal is to share and welcome the experience as it happened. This group rule lays the foundation for an innovative interpersonal climate in which the attitude of active, empathic, and compassionate listening is strengthened [35]. The main aim is to “bring benefits to one’s own and others’ experience” [31] and change the habit of critical, interpretative, or prejudicial listening.

An innovative aspect developed in the final meetings and pursued during the monthly follow-up sessions is the use of techniques based on imagination and guided visualization, using specific metaphors indicated by the Mindfulness masters (undercurrent, inn, and others metaphors) [31]. In this phase of the therapeutic work, when the meditation technique is more fluid and safer for patients, their psychoemotional management is deepened. Using new paradigms, new ways of analyzing and reflecting on psychoemotional experiences are developed, using the physical experience as a valid means of support for a functional relationship with the real experience “moment by moment” [31].

Mindfulness techniques implement awareness which aims to expand the ability to live an experience by focusing on the present; the ultimate meaning of this approach is to tune thoughts, experiences, sensations, and behaviors with what is really happening in patients’ everyday lives. This suggestion is linked to evidence that when arousal increases due to difficult situations, people tend to understand their experience and react on the basis of past learning. This frequently results in overestimation or devaluation of data, because people are not truly understanding what they are living. As a result, incorrect evaluations of experience, frequent misunderstandings or nonfunctional choices occur for patients who end up not being able to make informed and adequate choices, such as when they have to face and cope with oncological treatments and the surgical consequences on their body identity [8].

Even if individual psychological support meetings continue, the group path based on Mindfulness techniques ends with methods and goals strictly related to the experience of each patient. Monthly follow-up meetings are planned to maintain constant support in order to train the skills learned through Mindfulness meditation; teachings guide and encourage maintaining a bond with patients, in a group context. The main reason for continuing monthly group meetings is that the experience of cancer is characterized by continuous stress and uncertainty about the prognosis, which does not allow patients to consider themselves “cured”, but resilient. Consequently, as the patient engages in follow-up medical care that involves a periodic re-exposure to the trauma and associated fears, the experience of awareness and meditation benefits and supports the patient throughout their life [6,7,31].

### 2.5. Psychoncological Clinical History

The patient began psychoncological support care in the Day Hospital, during her first chemotherapy; B.A. expressed a mild mood and limited awareness of the disease. She manifested protective defense mechanisms of disengagement; the patient did not currently feel the need to engage in psychological support services. However, she agreed to continue the scheduled meetings.

During the individual sessions, B.A. recalled her personal and professional history, revealing a strong temperament (self-reliance) and rigid moral values. Her daily life was very challenging before the diagnosis; she traveled a great deal, and had intense relationships and working collaborations which gave her important gratifications. Analyzing the relationship with her family, B.A. reported a positive understanding and mutual support with her husband, and close parental relationships with all other family members, which are a source of fulfillment for her. She first described the diagnosis as a “bolt from the blue”, breaking into a family history that had not suffered any events of traumatic impact, up to that moment.

In conjunction with the onset of the treatment’s side effects, B.A. expressed a progressive psychoemotional demoralization and fragility, linked to fear and disorientation (affecting her role as mother, wife, daughter, and worker). As a result of these physical and psychological consequences, B.A. reported a reduction in her Quality of Life, together with intense emotionality, moments of strong discomfort, and uncertain care planning. These feelings were reactive to the difficult impact of the treatment’s side effects, which involved feeling alienated from her body and rejection of both aesthetic changes and her emerging weaknesses. She also assumed a more unregulated temperament, characterized by despair and loss of strength and psychological determination over time.

In this phase there was also a tendency toward isolation and sadness, which led the patient to limit activities outside the house; she also struggled to participate in clinical sessions, which were sometimes carried out via telemedicine. This allowed B.A. to stay home and feel safe, as she did not feel able to go to the hospital on her own. Therapeutic goals focused on listening and providing support to B.A. for emotional experiences, which took place continuously, destabilizing her.

The physical and emotional burdens experienced during this period led to a greater fragility of the patient’s ego. She appeared disoriented and engaged in regressive behaviors, requiring frequent assistance and comfort on many occasions. Her voice and attitude gave the impression of a “scared child”. It seemed that she felt the need to be protected and reassured about the future and the end of the disease, which she described as a “nightmare”.

The management of emotional experiences was also addressed during group therapy. In this setting, B.A. shared her emotional reactions with the other participants and established meaningful supportive relationships, which became a new impetus for her personal therapeutic work. In particular, the patient experienced “normalizing” her experience of the disease thanks to the mechanism of mirroring herself with other women and discussing issues such as her relationship with her body and sexuality. The experiences narrated by other patients with cancer allowed her to relinquish psychoemotional isolation and abandon the idea of being an abnormal person. All these processes let B.A. recognize herself as part of society again, as a woman, mother, worker, and sick person.

Subsequently, the psychoncologist met the patient and her husband (caregiver) in the ward, on the occasion of the mastectomy and reconstruction surgeries. The clinician took the opportunity to conduct a clinical interview with her husband, who appeared balanced and supportive, but expressed his own emotional issues due to his wife’s “intense suffering.” For this reason, the therapist helped him identify ways to support her, and to make it easier to manage home care. The main objective was to discuss the themes of intimacy and communication based on listening and accepting her pain and fear, without trying to resolve them; that is, letting B.A. express these experiences. Furthermore, the clinician aimed to increase moments of relaxation and fun together with their daughters, as well as moments dedicated to the couple, to provide opportunities to engage in typical daily experiences.

In the period subsequent to the mastectomy and reconstruction operation, B.A. reported a general improvement in physical conditions, which allowed her to resume normal daily activities. As a result, she manifested some recovery and began to take an interest in her personal and professional future again. For this reason, the therapist and the patient paused in-person sessions and engaged in telephone interviews. The goal was to resume psychological support interviews after the patient had recovered sufficient energy and had benefited from a period of rest, with the aim of stabilizing the resources acquired through individual and group therapies.

Afterwards, B.A. asked to resume supportive interviews at the beginning of radiotherapy, due to a reactivation of anxiety symptoms and insomnia that reawakened fragility and insecurity. In this period, due to painful physical symptoms that limited the quality of her life and autonomy, the patient tended to express anxious-depressive descriptions of herself and her relationships, which decreased only after the improvement of symptoms, and, consequently, of her autonomy.

Clinical sessions currently continue on a monthly basis, although the patient occasionally requests telephone interviews. They occur in moments in which she focuses on her fragility and uncertainty about the future. From a psychological point of view, B.A. maintains an unstable mood and a psychoemotional fragility, which she manages by practicing Mindfulness sessions at home and in groups during follow-up meetings.

In addition, she can count on the support of voluntary associations with whom she meets regularly, also as a volunteer, and on consistent and effective family emotional and moral support; she also slowly resumed her work activity.

## 3. Discussion

Many scientific studies [1,5,6] conducted around the world demonstrate the effectiveness of Mindfulness meditation practice in (1) increasing people’s awareness of their inner states, (2) modifying people’s relationships with themselves by decreasing aspects of criticality and devaluation, (3) facilitating the ability to cultivate affectionate, compassionate, and supportive views of their own experiences including emotions, thoughts, behaviors, and sensations, and (4) developing compassion and extending this feeling to the surrounding world.

The ultimate goal of Mindfulness is the desire to obtain serenity, peace and hope. This process fosters conducting relationships based on mutual respect and understanding, and a decrease in the tendency towards isolation and conflict. Mindfulness also builds resilience by improving strategies to reduce stressful psychological and emotional experiences, and prevent their recurrence in the future.

Previous studies [6] show that Mindfulness meditation techniques can contribute to a significant attenuation of the impact of psychological distress due to the stressful and uncertain path associated with cancer. This practice, in fact, allows patients to identify new functional coping strategies to improve their Quality of Life and psychological well-being. This psychophysical balance appears even more relevant in cases where the disease becomes more chronic, and patients and family members have to deal with a constant threat that affects their lifestyle and planning. This case report intends to promote clinical and experimental study in order to obtain more evidence regarding the usefulness and efficacy of the integrated use of individual and group therapy as well as the efficacy of Mindfulness pathways on the psychosocial management of breast cancer. At our Institute we observe different outcomes of this type of treatment, such as the duration and quality of cognitive and emotional empowerment; therefore, we wish to carry out such research, the data from which may currently be classified as insights based on the clinical efficacy of the treatments carried out.

The validity of this practice was tested in many studies, which show that it provides effective support in the management of symptoms, especially psychological issues [13,31].

After group treatment, B.A. found the motivation to return to her daily activities again, although it initially seemed far outside the realm of her possibilities. Therefore, the theoretical hypothesis that Mindfulness meditation facilitates the management of fatigue, which normally limits the maintenance of daily activities and interpersonal relationships, was confirmed in this case [23]. According to previous investigations, this effect on fatigue may result from an increase in oxygen levels in muscles and blood, an increase in energy, and the positive influence of the emotional aspects that meditation instills in patients [36,37]. Thus, the initial hypothesis that awareness improves the coping skills of cancer patients seems to be supported by the fact that B.A. progressively expressed a better ability to cope, adapt, and respond to both symptoms and difficulties associated with the disease.

One of the most common hypotheses regarding breast cancer psychosocial treatment is that awareness increases cognitive and behavioral coping [13,38,39]. Even in the follow-up, better coping strategies oriented to the problem (instrumental support, active coping, and planning) and to emotions (acceptance, positive reprogramming, and use of emotional support) emerged. These data appear relevant as the first type of coping is to operate actively on the causes of stress, while the second type supports people’s self-reflection and the expression and processing of their emotions [40,41,42].

In conclusion, it is possible to focus on B.A.’s Quality of Life, which was strengthened from multiple points of view; she resumed her life planning in both social and work realms, her sleep-wake rhythm became more regulated, and there was better management of related-traumatic symptoms such as arousal and defensive attitudes. In addition, B.A. reported improvements related to both the physical (pain, inflammation, fatigue, and sleep symptoms) and psychological (anxiety, depression, emotional regulation, and spiritual) components of Quality of Life [39,43,44]. The hypothesis that this improvement in Quality of Life is due to individual and group psychoncological interventions can be supported by the literature, which shows that Mindfulness-based programs that also include meditation, relaxation, and daily practice, may lead to a significant improvement in psychological well-being [45]. We hope this hypothesis will be supported by future research on experimental and control groups for the purpose of generating evidence-based scientific data.

## 4. Research Method

The method employed in the present paper is the Case Study, commonly used in the social and health sciences. The Case Study can be defined as an empirical research method used to investigate a contemporary phenomenon, focusing on the dynamics of the case in its real life context [46]. It is used to describe all those unique situations or phenomena which need more information within one field of research. This methodology is similar to qualitative research, since it focuses on the exhaustive study of a phenomenon and not on the statistical analysis of existing data. Traditionally, the case study begins with the presentation of the patient and his/her problems (physical, emotional or sensory symptoms, as well as thoughts, feelings and perceptions related to the symptoms); then, it focuses on the description of the intervention used to help the patient (psychoanalytic, cognitive-behavioral or humanistic approach) [47]. In this specific case report, a combination of psychological counseling and Mindfulness meditation techniques was used.

## 5. Conclusions

The hypothesis proposals in this case report suggest that Mindfulness can support psychological and biological improvements in cancer patients, especially in a group setting. Therefore, Mindfulness meditative practice can be offered as a valid approach to reduce depressive distress symptoms and strengthen coping strategies, and, consequently, facilitate the survival of women with breast cancer.

Interestingly, our clinical results suggest that individual psychological support can be combined with group therapeutic work, which includes psychosocial support. By engaging in group intervention, patients may be able to develop a new self-awareness, which allows generalizable cognitive, behavioral, and emotional empowerment in different contexts of daily life.

Future developments related to the findings of this project might include longitudinal studies, which are generally useful for investigating the outcomes that emerge during the progression and survival phases of the disease. In addition, period surveys (e.g., distress, Quality of Life, coping) might be used to track quantitative results. On a larger scale, it may be interesting to evaluate a larger group of patients who receive a combination of individual and group Mindfulness training, and to compare their outcomes with a control group. Finally, future research can also explore the differences between patients who undertake Mindfulness group activities and those who benefit from classic psychological support groups.

## Data Availability

Not applicable.
